# Statistical significance of cis-regulatory modules

**DOI:** 10.1186/1471-2105-8-19

**Published:** 2007-01-22

**Authors:** Dustin E Schones, Andrew D Smith, Michael Q Zhang

**Affiliations:** 1 Cold Spring Harbor Laboratory, Cold Spring Harbor, NY, 11724, USA; 2 Department of Physics and Astronomy, Stony Brook University, Stony Brook, NY 11790, USA

## Abstract

**Background:**

It is becoming increasingly important for researchers to be able to scan through large genomic regions for transcription factor binding sites or clusters of binding sites forming *cis*-regulatory modules. Correspondingly, there has been a push to develop algorithms for the rapid detection and assessment of *cis*-regulatory modules. While various algorithms for this purpose have been introduced, most are not well suited for rapid, genome scale scanning.

**Results:**

We introduce methods designed for the detection and statistical evaluation of *cis*-regulatory modules, modeled as either clusters of individual binding sites or as combinations of sites with constrained organization. In order to determine the statistical significance of module sites, we first need a method to determine the statistical significance of single transcription factor binding site matches. We introduce a straightforward method of estimating the statistical significance of single site matches using a database of known promoters to produce data structures that can be used to estimate *p*-values for binding site matches. We next introduce a technique to calculate the statistical significance of the arrangement of binding sites within a module using a max-gap model. If the module scanned for has defined organizational parameters, the probability of the module is corrected to account for organizational constraints. The statistical significance of single site matches and the architecture of sites within the module can be combined to provide an overall estimation of statistical significance of *cis*-regulatory module sites.

**Conclusion:**

The methods introduced in this paper allow for the detection and statistical evaluation of single transcription factor binding sites and *cis*-regulatory modules. The features described are implemented in the Search Tool for Occurrences of Regulatory Motifs (STORM) and MODSTORM software.

## Background

The identification of transcription factor binding sites and *cis*-regulatory modules is a crucial step in the study of gene regulation. Transcription factor binding sites often occur together in clusters as *cis*-regulatory modules and these modules can regulate the transcription of genes 100 kilobases (kb) or further from the module sites [1,2]. To study the regulation of transcription *in silico*, it has become necessary to scan large intergenic regions for occurrences of these modules. When scanning through large intergenic regions, it is important to be able to evaluate the statistical significance of predicted module sites in order to distinguish likely modules for experimental validation. The methods we introduce in this paper allow for the rapid detection of *cis*-regulatory module sites as well as an estimation of their statistical significance. These tools will be of great use to any researcher wishing to scan large intergenic regions for potential *cis*-regulatory modules.

The *p*-value corresponding to a given match score for a transcription factor binding site is the probability of observing a match score at least as great for a randomly generated site. We start by describing a method for the accurate estimation of statistical significance of single binding sites. This method utilizes an extensive database of known human, mouse and rat transcription start sites, the CSHL mammalian promoter database (CSHLmpd) [3] to tabulate frequencies of occurrences for all *k*-mers in known sequences. This information is then used to calculate *p*-values that correspond to particular thresholds for matrices as well as to calculate the threshold of occurrence corresponding to a given *p*-value. If a number of binding sites have been predicted and a position frequency matrix (PFM) that corresponds to these binding sites is known, this technique can be used to obtain a *p*-value for the predicted sites. Conversely, if the desired *p*-value is known for a given matrix, the threshold corresponding to this *p*-value can be calculated and this can be used to find the sites in sequences. One major advantage of using an extensive database of known sequence regions to estimate *p*-values is that the result will not depend on the sample data and therefore results obtained from different motif-finding or scanning algorithms can be compared using *p*-values. Although previous work has addressed the problem of estimating the statistical significance of binding site matches, these methods were forced to either assume uniform nucleotide frequencies for all sequences tested or to look at distributions of scores across random sequences to be computationally feasible. The novel methods we present here utilize known DNA sequence in an efficient manner to avoid any prior assumptions on nucleotide distribution in regulatory DNA sequences. This allows us to make more accurate estimates on statistical significance of binding site predictions.

After describing the estimation of *p*-values for single transcription factor binding sites, we show how the single site *p*-values can be combined with a clustering probability to determine the overall module probability. We consider two separate models of *cis*-regulatory modules, modules with constrained organization and modules without constrained organization. Modules without organizational constraints are simply a cluster of individual binding sites with the only parameters of the model being the *p*-values of the individual sites and the clustering *p*-value. The clustering probability in this model is calculated using a max-gap model. We also consider modules that have prescribed organizational constraints. The organizational parameters we consider are the order, spacing and orientation of motifs in the module. For modules with organizational constraints, the module probability is calculated using a max-gap model, and then adjusted for the organizational parameters.

## Results

### Statistical significance of single sites

#### Previous work on statistical significance of single sites

Many approaches to obtain *p*-values for motif site matches using position weight matrices (PWMs) have been introduced [4-12]. We now briefly review the original works in this field, and touch upon some of the more recent work that has extended these original methods.

The work of Staden is the original work on calculating probabilities for finding motif matches in DNA sequence [4]. The method outlined by Staden uses probability generating functions to calculate the theoretical probability of finding motif matches. Much of the existing work on calculating probabilities of motif matches is built directly on Staden's work. Claverie showed that given the base composition of random sequence, one can compute the expected distribution of random scores for each individual position and, iteratively, the expected distribution of the total score [5]. This distribution of scores is close to a Gaussian, as in Staden's work, and the expected best matching score in each sequence follows an extreme value distribution. The software program NMksite allows for the calculation of statistical significance given a PFM and a threshold [6]. Beckstette *et al*. [11], introduced the ESAsearch algorithm which assesses the statistical significance of binding site matches using a method based on Staden's work. Baily and Gribskov introduced a method to estimate the statistical significance of motif matches based on Fisher's "omnibus" procedure for combining one-sided statistical tests [7]. Hertz and Stormo introduced two methods to approximate the significance of motif matches, one based on large-deviation theory and one purely numerical [8]. Nagarajan *et al*. extended the numerical method of Hertz and Stormo using a cyclic shifted Fast Fourier Transform [12].

#### Obtaining match *p*-values from database frequencies

Assuming the sequences being scanned are a sample from a known population of sequences, we can query that population directly to obtain the *p*-value associated with any match score. For example, suppose we are searching a set *S *of 10 kb sequences immediately upstream of the transcription start site (TSS) of a set of human genes. The population *D *would be the entire set of 10 kb upstream regions in the human genome. For a scoring matrix *M*, and a match score *t*, the probability that a randomly selected site from *S *scores at least *t *when matched with *M *is

Pr⁡ (score(M,s)≥t|s∈S=|{s∈D:score(M,s)≥t}||ND|,     (1)
 MathType@MTEF@5@5@+=feaafiart1ev1aaatCvAUfKttLearuWrP9MDH5MBPbIqV92AaeXatLxBI9gBaebbnrfifHhDYfgasaacH8akY=wiFfYdH8Gipec8Eeeu0xXdbba9frFj0=OqFfea0dXdd9vqai=hGuQ8kuc9pgc9s8qqaq=dirpe0xb9q8qiLsFr0=vr0=vr0dc8meaabaqaciaacaGaaeqabaqabeGadaaakeaacyGGqbaucqGGYbGCcqqGGaaicqGGOaakcqqGZbWCcqqGJbWycqqGVbWBcqqGYbGCcqqGLbqzcqGGOaakcqWGnbqtcqGGSaalcqWGZbWCcqGGPaqkcqGHLjYScqWG0baDcqGG8baFcqWGZbWCcqGHiiIZcqWGtbWucqGH9aqpdaWcaaqaaiabcYha8jabcUha7jabdohaZjabgIGiolabdseaejabcQda6iabbohaZjabbogaJjabb+gaVjabbkhaYjabbwgaLjabcIcaOiabd2eanjabcYcaSiabdohaZjabcMcaPiabgwMiZkabdsha0jabc2ha9jabcYha8bqaaiabcYha8jabd6eaonaaBaaaleaacqWGebaraeqaaOGaeiiFaWhaaiabcYcaSiaaxMaacaWLjaWaaeWaaeaacqaIXaqmaiaawIcacaGLPaaaaaa@6B0E@

where *S *and *D *are treated as the sets of sites they contain, and *N*_*D *_is the number of possible sites in *D*. The ideal way to evaluate Equation 1 is to scan all sequences in *D *and count the sites with scores at least as great as *t*. In most applications, however, *D *is prohibitively large and this ideal solution is impractical.

**Figure 1:**
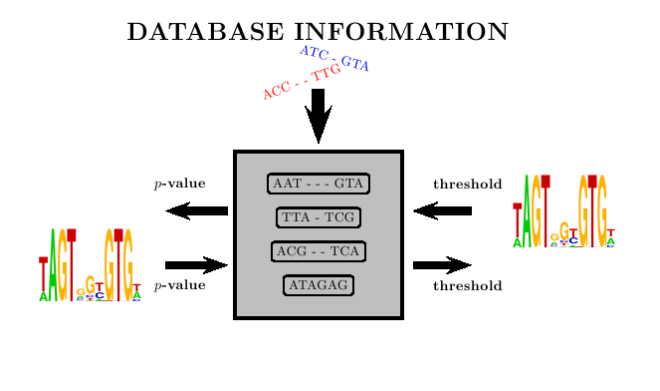
**Converting between *p*-values and match scores using (*g*, *k*)-tables**. Based on a sequence database, a (*g*, *k*)-table encodes sufficient information to calculate match score *p*-values, or produce a score cutoff corresponding to a given *p*-value. Details of this process are described in the text.

We describe here a technique that preprocesses a sequence database to create a summary data structure encoding sufficient information to accurately estimate the probability described in Equation 1. We refer to these summary data structures as (*g*, *k*)-tables, because they tabulate all *g*-spaced *k*-mers (defined below) in the sequence database. Given a matrix and a cutoff score, a (*g*, *k*)-table allows rapid estimation of the *p*-value for that score. Similarly, the (*g*, *k*)-table allows a cutoff score associated with a given *p*-value to be computed. This process is illustrated in Figure 1.

#### Preprocessing sequence databases as (*g*, *k*)-tables

A *g*-spaced *k*-mer is a sequence segment that consists of *k*/2 contiguous nucleotides, a space of width *g *following position *k*/2, and the next *k*/2 contiguous nucleotides starting at position *k*/2 + *g *+ 1 (enforcing *k *to be even). As an example, the 5-spaced 6-mer ACGTCA occurs in the following sequence beginning at the fourth position:

TAGACGTTATGTCAA

Although it is possible to construct (*g*, *k*)-tables from other formations of words, this format of a *g*-spaced *k*-mer can adequately represent the majority of binding sites present in the TRANSFAC [13] and Jaspar [14] databases. A sequence database *D *may be either a single long sequence or multiple sequences. Both cases are treated similarly. The (*g*, *k*)-table for database *D *contains the number of times each (*g'*-spaced *k*-mer occurs in a sequence of *D*, for 0 ≤ *g' *≤ *g*. For a given word *w *of width *k *and containing *g *spaces, the (*g*, *k*)-table documents the number of times *w *occurs in *D *with at most *g *central spaces. For larger values of *k *and *g*, a (*g*, *k*)-table contains more information about *D*, providing more accurate *p*-value estimates with more of the positions in a matrix considered exactly. However, the total size of the (*g*, *k*)-table will be larger.

#### Calculating expected frequencies of long words

To calculate the expected frequency of words longer than the words tabulated from the database, we first look for the most informative positions in the matrix, with the information content of positions in the matrix defined as in [15]. If the tabulated frequencies are of size *k*, we find the most informative positions in the matrix that are in the format of a *g*-spaced *k*-mer: *k*/2-gap-*k*/2. All *k*-mers that can be generated from these positions are enumerated using a branch and bound algorithm. The frequency of each *k*-mer above threshold is then looked up in the (*g*, *k*)-table. To estimate the expected frequencies of the longer words we use the nucleotide content of the database to create a Markov model of order *k*/2. With this, the consecutive positions of the matrix that are not part of the *k *most informative positions contribute to the expected frequency in a way that is proportional to their frequency in the promoter database.

#### Converting between *p*-values and scores

Given a scoring matrix *M*, and a match score *t*, let *W *(*M*, *t*) be the set of all words *w *such that score(*M*, *w*) ≥ *t*. Define *N*_*est *_(*w*, *D*) as the estimated number of times the word *w *occurs in sequences of *D*. We estimate the probability from Equation 1 as

Pr⁡ (score(M,s)≥t|s∈D)≈1ND∑w∈W(M,t)Nest(w,D).
 MathType@MTEF@5@5@+=feaafiart1ev1aaatCvAUfKttLearuWrP9MDH5MBPbIqV92AaeXatLxBI9gBaebbnrfifHhDYfgasaacH8akY=wiFfYdH8Gipec8Eeeu0xXdbba9frFj0=OqFfea0dXdd9vqai=hGuQ8kuc9pgc9s8qqaq=dirpe0xb9q8qiLsFr0=vr0=vr0dc8meaabaqaciaacaGaaeqabaqabeGadaaakeaacyGGqbaucqGGYbGCcqqGGaaicqGGOaakcqqGZbWCcqqGJbWycqqGVbWBcqqGYbGCcqqGLbqzcqGGOaakcqWGnbqtcqGGSaalcqWGZbWCcqGGPaqkcqGHLjYScqWG0baDcqGG8baFcqWGZbWCcqGHiiIZcqWGebarcqGGPaqkcqGHijYUdaWcaaqaaiabigdaXaqaaiabd6eaonaaBaaaleaacqWGebaraeqaaaaakmaaqafabaGaemOta40aaSbaaSqaaiabdwgaLjabdohaZjabdsha0bqabaGccqGGOaakcqWG3bWDcqGGSaalcqWGebarcqGGPaqkaSqaaiabdEha3jabgIGiolabdEfaxjabcIcaOiabd2eanjabcYcaSiabdsha0jabcMcaPaqab0GaeyyeIuoakiabc6caUaaa@62A3@

We now describe how, for a given word *w*, we obtain an accurate value for *N*_*est *_(*w*, *D*) using a (*g*, *k*)-table. We first identify the *k *positions in *M *with the greatest information content, under the restriction that those positions must match the format of a *g*-spaced *k*-mer. Let *w' *be the *g*-spaced *k*-mer in *w *corresponding to the aforementioned most informative positions in *M*. Using the (*g*, *k*)-table, we obtain the exact number of times *w' *occurs in *D*. This number of occurrences is then multiplied by the probability of observing the nucleotides at remaining positions in *w *given the nucleotides at positions in *w'*, as described above.

Implementing this technique, for a given matrix, requires enumerating all words with a match score greater than the specified cutoff. Such enumeration is feasible for the majority of binding site matrices. If a *p*-value is given instead of a threshold, the process is reversed: the *p*-value is converted into an occurrence frequency, and the (*g*, *k*)-table is used to estimate a score threshold for which we will observe that occurrence frequency in the database. When scanning sequences, each site constitutes a distinct trial, and therefore, the *p*-values must be corrected for multiple testing. The simple Bonferroni correction [16], which assumes independence between trials, may be used in this case. Any dependencies introduced by autocorrelation are accounted for by the counts tabulated in the (*g*, *k*)-tables: autocorrelating words will have greater counts in the database, and this is directly reflected in the (*g*, *k*)-table.

#### Accuracy of (*g*, *k*)-table calculations

To assess our ability to determine *p*-values of binding site predictions using (*g*, *k*)-tables, we carried out the following tests. Tables for two different size promoters: small (-500 to +100 w.r.t TSS) and large (-5000 to +500 w.r.t. TSS), and two sizes of *k*-mers: 6-mers and 8-mers, were constructed for all human promoters in CSHLmpd. The subset of TRANSFAC (v9.3, [13]) matrices described used for testing was all vertebrate matrices with integer counts.

Each matrix in the subset was scanned on both of the human promoter sets and thresholds for site matches were set at a functional depth of 0.9. A functional depth (described in the Methods section) of 0.9 was used here because all matrices have a reasonable match threshold corresponding to this functional depth. The empirical *p*-value corresponding to this functional depth was then calculated as the number of site matches divided by the total number of windows of the size of the matrix contained in the particular promoter set. The matrix threshold corresponding to a functional depth of 0.9 was then converted to a *p*-value using the appropriate (*g*, *k*)-table and an error was calculated as the difference between the empirical *p*-value calculated as above and the *p*-value calculated using the (*g*, *k*)-table. The average error for all matrices tested for both sizes of promoters and *k*-mers is shown in Table 1.

**Table 1:** Error in the estimation of *p*-values using (*g*, *k*)-tables.

**Test**	***k*-mer size**	**Promoter Set**	**Error (Ave ± S.D.)**
(*g*, *k*)-table	6	small	3.53e-5 ± 1.87e-4
		large	2.65e-5 ± 1.04e-4
	8	small	1.84e-5 ± 6.57e-5
		large	1.16e-5 ± 4.35e-5

ESAsearch		small	1.23e-4 ± 5.96e-4
		large	1.61e-4 ± 7.68e-4

Errors in the estimation of *p*-values using (*g*, *k*)-tables of various sequence sets and also the ESAsearch program [11,56]. The error in the calculation of *p*-values using (*g*, *k*)-tables decreases as larger *k*-mers and larger sequences are used to build the (*g*, *k*)-tables.

We also wanted to measure the error created with the approach to *p*-value calculation as implemented in the ESAsearch program. To test this software, we first calculated an empirical *p*-value for each matrix in the manner described above. This *p*-value was then used as an input parameter for ESAsearch to scan for sites with at least this probability. The resulting number of sites found by ESAsearch was used to calculate the empirical *p*-value resulting for the program and the error in the process was calculated as the difference in the two *p*-values. The average errors produced by the ESAsearch algorithm are listed in Table 1.

The error in the calculations using the (*g*, *k*)-tables decreases as we use larger promoters and larger *k*-mers to construct the (*g*, *k*)-tables, which is expected. As more information is used in the calculation, the errors involved will be reduced. More suggestions on the proper selection of sequences to construct a (*g*, *k*)-table are included in the Discussion section. We also tested the NMksite software [6] in its ability to carry out this procedure. For the two cases it was tested on (both small and large promoters), out of the 524 matrices that were tested, NMksite could not return thresholds for *p*-values small enough for 148 and 132 of the matrices respectively. This is due to the fact that NMksite produces a distribution of thresholds and *p*-values and if the desired *p*-value is smaller than any of the *p*-values listed by NMksite, the user is unable to get a corresponding threshold. If we ignored the matrices NMksite could not determine thresholds for, the errors for the small and large promoter sets were 1.30e-2 and 1.35e-2 respectively, which are much larger than the errors introduced by either the ESAsearch or (*g*, *k*)-table method.

### Statistical significance of module sites

As mentioned previously, it is often the case that binding sites occur together in clusters as *cis*-regulatory modules and these modules can occur in regions very far from the genes they regulate. In this section, we extend the method described above for the estimation of statistical significance of single binding sites to the statistical significance of modules.

To assess the statistical significance of gene clusters, Hoberman *et al*. [17,18] developed statistics for gene clusters based on a max-gap parameter, which is defined as the maximum distance allowed between any two genes in a cluster. In the following sections, we outline how the max-gap technique can be extended to assessing the statistical significance of clusters of transcription factor binding sites. Once we have estimated the statistical significance of the clustering of motif sites, we can combine *p*-values for individual sites with the clustering *p*-value to get an overall significance estimation for *cis*-regulatory modules.

#### Previous work on scanning for *cis*-regulatory modules

The algorithms that have been introduced to find *cis*-regulatory modules can be divided into four types of algorithms: (1) algorithms for *de novo *module discovery, (2) algorithms that attempt to cluster binding sites found with traditional methods into modules, (3) algorithms that use phylogenetic information and (4) algorithms that scan for a pre-defined module profile. Algorithms for *de novo *discovery of modules include ModuleSearcher [19], Co-Bind [20], Bioprospector [21], MITRA [22], dyad search [23], CisModule [24], EMCModule [25] and the Gibbs Module Sampler [26]. Algorithms that combine single sites together to form modules include the logistic regression analysis method introduced by Wasserman and Fickett [27], MSCAN [28,29], COMET [30,31], MCAST [32], Composite Module Analysis [33], Ahab [34], ModuleFinder [35] and methods based on estimating the clustering significance of single sites under a Poisson distribution model of motif sites used by Lifanov *et al*. [36] and Wagner [37-39]. In addition to these methods, a number of recent algorithms have used conservation to try to predict *cis*-regulatory modules [40-44]. Algorithms designed specifically for scanning include CisModScan [45], Target Explorer [46] and ModuleScanner [19]. We will discuss these algorithms further when comparing their performance to our method.

The original work by Wasserman and Fickett [27] took into account the overall organization of a module in that their technique has the ability to learn the order of the motifs in a module and the method used by Klingenhoff *et al*. considered the distances between single sites in a module [47]. A few of the more recent algorithms for *de novo *module discovery have also begun to take into account the organization of modules. For example, the algorithms introduced by Gupta and Liu [25] and Thompson *et al*. [26] take into account the distances between motif sites in a module. These methods, however, are not built for scanning through sequences for defined modules but rather for *de novo *discovery of modules. None of the existing algorithms that allow for identifying and estimating the statistical significance of module sites allow the user to search for modules with defined organization.

#### Statistical significance of modules without constrained organization

**Figure 2:**
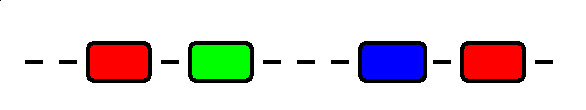
**Max-gap cluster – module without organizational constraints**. A max-gap cluster of motifs as a module without organizational constraints. The length of the sequence (*l*) is 16 bases, the number of motifs (*m*) is four, the widths of each motif (*w*_*i*_) is two bases and the max-gap (*g*) is three bases.

In this section we deal with the calculation of statistical significance of motif clusters with no consideration given to overall module organization. Hoberman *et al*. [17,18] considered the clustering of a chain of genes where genes were treated as points in a chain. Therefore, they did not have to deal with the lengths of genes or the lengths of gaps between genes in a cluster. For our situation, we are considering motifs in a sequence window and the lengths of motifs are very important. Our treatment of the max-gap statistics diverges from that of Hoberman *et al*. due to this. An example of a max-gap cluster of *m *binding sites of width *w *in a sequence of length *l *with a max-gap *g *can be seen in Figure 2.

#### Counting arrangements

The number of ways of creating a max-gap cluster of *m *motifs, a maximum gap of *g *and total length *l *is equivalent to rolling to *m *- 1 dice, with faces numbered 0 to *g*, such that the sum of their faces is equal to *l *- ∑_*i *_*w*_*i*_, where 0 <*i *≤ *m*. This is, in turn, equivalent to rolling to *m *- 1 dice with faces numbered 1 to *g *+ 1 with the sum of their faces equal to *l *- ∑_*i *_(*w*_*i *_- 1) - 1. Using this analogy, Hoberman *et al*. [17,18] derived an expression for the number of ways to construct a max-gap cluster of *m *genes anywhere in a sequence of length *l*. When considering motifs with widths, the expression

E(m,w,g,l)=m!∑k=0⌊l−∑i(wi−1)−mg+1⌋(−1)k(m−1k)(l−∑i(wi−1)−k(g+1)m),     (2)
 MathType@MTEF@5@5@+=feaafiart1ev1aaatCvAUfKttLearuWrP9MDH5MBPbIqV92AaeXatLxBI9gBaebbnrfifHhDYfgasaacH8akY=wiFfYdH8Gipec8Eeeu0xXdbba9frFj0=OqFfea0dXdd9vqai=hGuQ8kuc9pgc9s8qqaq=dirpe0xb9q8qiLsFr0=vr0=vr0dc8meaabaqaciaacaGaaeqabaqabeGadaaakeaacqWGfbqrcqGGOaakcqWGTbqBcqGGSaalcqWG3bWDcqGGSaalcqWGNbWzcqGGSaalcqWGSbaBcqGGPaqkcqGH9aqpcqWGTbqBcqGGHaqidaaeWbqaamaabmaabaGaeyOeI0IaeGymaedacaGLOaGaayzkaaWaaWbaaSqabeaacqWGRbWAaaaabaGaem4AaSMaeyypa0JaeGimaadabaWaayWaaeaadaWcaaqaaiabdYgaSjabgkHiTmaaqababaGaeiikaGIaem4DaC3aaSbaaWqaaiabdMgaPbqabaWccqGHsislcqaIXaqmcqGGPaqkcqGHsislcqWGTbqBaWqaaiabdMgaPbqab4GaeyyeIuoaaSqaaiabdEgaNjabgUcaRiabigdaXaaaaiaawcp+caGL7Jpaa0GaeyyeIuoakmaabmaabaqbaeqabiqaaaqaaiabd2gaTjabgkHiTiabigdaXaqaaiabdUgaRbaaaiaawIcacaGLPaaadaqadaqaauaabeqaceaaaeaacqWGSbaBcqGHsisldaaeqaqaaiabcIcaOiabdEha3naaBaaaleaacqWGPbqAaeqaaOGaeyOeI0IaeGymaeJaeiykaKIaeyOeI0Iaem4AaSMaeiikaGIaem4zaCMaey4kaSIaeGymaedaleaacqWGPbqAaeqaniabggHiLdGccqGGPaqkaeaacqWGTbqBaaaacaGLOaGaayzkaaGaeiilaWIaaCzcaiaaxMaadaqadaqaaiabikdaYaGaayjkaiaawMcaaaaa@7D50@

counts the number of ways to form a max-gap cluster of *m *factors with widths *w*_*i*_, in a sequence of length *l *and a max-gap parameter *g*. The differences in this expression and the expression derived by Hoberman *et al*. are first, in Hoberman *et al*., the sum of all the gaps was equal to *l *- 1 while here the sum of all the gaps is equal to *l *- ∑_*i *_(*w*_*i *_- 1) - 1 and second, we multiply the entire expression by *m*! because a group of factors in a unique order qualifies as a unique module.

#### Probabilities for the occurrence of clusters

With the ability to enumerate all possible ways to place *m *factors with widths *w*_*i *_in a sequence of length *l *such that the maximum possible gap allowed between any two factors is *g *we can calculate a *p*-value for the max-gap configuration. Under the null hypothesis that each site is uniformly distributed in the sequence, the *p*-value for the max-gap configuration is

Pr⁡(gap≤g|m,w,l)=E(m,w,g,min⁡(l,∑i=1mwi+(m−1)g))(1−∑i(wi−1)m).     (3)
 MathType@MTEF@5@5@+=feaafiart1ev1aaatCvAUfKttLearuWrP9MDH5MBPbIqV92AaeXatLxBI9gBaebbnrfifHhDYfgasaacH8akY=wiFfYdH8Gipec8Eeeu0xXdbba9frFj0=OqFfea0dXdd9vqai=hGuQ8kuc9pgc9s8qqaq=dirpe0xb9q8qiLsFr0=vr0=vr0dc8meaabaqaciaacaGaaeqabaqabeGadaaakeaacyGGqbaucqGGYbGCcqGGOaakcqqGNbWzcqqGHbqycqqGWbaCcqGHKjYOcqWGNbWzcqGG8baFcqWGTbqBcqGGSaalcqWG3bWDcqGGSaalcqWGSbaBcqGGPaqkcqGH9aqpdaWcaaqaaiabdweafjabcIcaOiabd2gaTjabcYcaSiabdEha3jabcYcaSiabdEgaNjabcYcaSiGbc2gaTjabcMgaPjabc6gaUjabcIcaOiabdYgaSjabcYcaSmaaqadabaGaem4DaC3aaSbaaSqaaiabdMgaPbqabaGccqGHRaWkcqGGOaakcqWGTbqBcqGHsislcqaIXaqmcqGGPaqkcqWGNbWzcqGGPaqkcqGGPaqkaSqaaiabdMgaPjabg2da9iabigdaXaqaaiabd2gaTbqdcqGHris5aaGcbaWaaeWaaeaafaqabeGabaaabaGaeGymaeJaeyOeI0YaaabeaeaacqGGOaakcqWG3bWDdaWgaaWcbaGaemyAaKgabeaakiabgkHiTiabigdaXiabcMcaPaWcbaGaemyAaKgabeqdcqGHris5aaGcbaGaemyBa0gaaaGaayjkaiaawMcaaaaacqGGUaGlcaWLjaGaaCzcamaabmaabaGaeG4mamdacaGLOaGaayzkaaaaaa@768E@

The numerator in this equation is given by Equation 2 with the *m*! factor canceled by the denominator. The denominator in this equation is the number of ways to place *m *non-overlapping motifs in a sequence of length *l *(see Additional file 1), with the *m*! factor canceled by the numerator.

#### Statistical significance of modules with constrained organization

**Figure 3:**
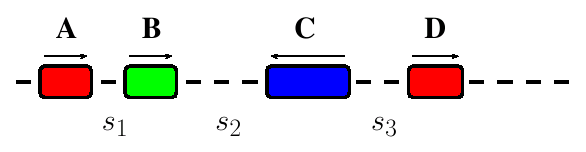
**Max-gap cluster – module with organizational constraints**. Max-gap cluster of motifs as a module with organizational constraints. There are four motifs in this module that must occur in the order A,B,C,D. The spacings between the motifs are defined as *s*_*x *_and the orientations of the motifs are labeled with arrows above the motifs.

Here we extend the treatment of modules without organizational constraints to deal with the organizational parameters of the ordering of motifs, the spacing between the motifs and the orientation of the motifs in a module. Any or all of these organizational parameters can be specified when searching for a given module. In this section we outline methods to calculate statistical significance of module sites with any or all of these organizational parameters specified. In each case, the overall probability of the cluster with no organization is first calculated, and then this probability is corrected for the organizational constraints. An example of a module with specified organizational parameters can be seen in Figure 3. We note that a smaller module with organization can exist within a larger module without organization.

#### Modules with order specified

We again define *m *as the number of motifs, *g *as the max-gap parameter and *l *as the total length of the window. The total number of permutations of *m *factors is simply *m*!. Therefore, if the order of the factors in a module is specified, along with a max-gap parameter, the probability of the module is:

Pr(gap ≤ *g*|{*m*, *w*, *l*}, *r*) = Pr(gap ≤ *g*, |*m*, *w*, *l*) *P *(*r*),

where Pr(gap ≤ *g*|*m*, *w*, *l*) is given by Equation 3 and,

P(r)=1m!.     (4)
 MathType@MTEF@5@5@+=feaafiart1ev1aaatCvAUfKttLearuWrP9MDH5MBPbIqV92AaeXatLxBI9gBaebbnrfifHhDYfgasaacH8akY=wiFfYdH8Gipec8Eeeu0xXdbba9frFj0=OqFfea0dXdd9vqai=hGuQ8kuc9pgc9s8qqaq=dirpe0xb9q8qiLsFr0=vr0=vr0dc8meaabaqaciaacaGaaeqabaqabeGadaaakeaacqWGqbaucqGGOaakcqWGYbGCcqGGPaqkcqGH9aqpdaWcaaqaaiabigdaXaqaaiabd2gaTjabcgcaHaaacqGGUaGlcaWLjaGaaCzcamaabmaabaGaeGinaqdacaGLOaGaayzkaaaaaa@39CE@

#### Modules with spacing specified

The correction for the probability of a module site when the spacing between any two pairs of factors is known can be calculated as

Pr(gap ≤ *g*|{*m*, *w*, *l*}, *r*, *s*) = Pr(gap ≤ *g*, |*m*, *w*, *l*) *P *(*r*) *P *(*s*),

where Pr(gap ≤ *g*|*m*, *w*, *l*) is given by Equation 3, *P *(*r*) is given by Equation 4 and

P(s)=∏i=1msspanig,     (5)
 MathType@MTEF@5@5@+=feaafiart1ev1aaatCvAUfKttLearuWrP9MDH5MBPbIqV92AaeXatLxBI9gBaebbnrfifHhDYfgasaacH8akY=wiFfYdH8Gipec8Eeeu0xXdbba9frFj0=OqFfea0dXdd9vqai=hGuQ8kuc9pgc9s8qqaq=dirpe0xb9q8qiLsFr0=vr0=vr0dc8meaabaqaciaacaGaaeqabaqabeGadaaakeaacqWGqbaucqGGOaakcqWGZbWCcqGGPaqkcqGH9aqpdaqeWbqaamaalaaabaGaee4CamNaeeiCaaNaeeyyaeMaeeOBa42aaSbaaSqaaiabdMgaPbqabaaakeaacqWGNbWzaaaaleaacqWGPbqAcqGH9aqpcqaIXaqmaeaacqWGTbqBdaWgaaadbaGaem4Camhabeaaa0Gaey4dIunakiabcYcaSiaaxMaacaWLjaWaaeWaaeaacqaI1aqnaiaawIcacaGLPaaaaaa@47A4@

where *m*_*s *_is the number of spacings which are defined in the organization and span_*i *_is the size of the allowed span between the two factors flanking the *i*^th ^spacing (e.g. span_*i *_= 25 if the spacing between motif A and motif B is known to be between 10 and 35 bases). In this situation we have also corrected for the order of the motifs in the module being specified. In general, when the spacing between two factors is defined, the order of the factors will be defined as well.

#### Modules with strands specified

If the strands of the motifs in the module are specified, then the final probability of the module is simply multiplied by 1/2 for every motif that has a specified strand. Therefore, if the organization of a module has specified strands, the final probability for the module is

Pr(gap ≤ *g*|{*m*, *w*, *l*}, *d*) = Pr(gap ≤ *g*|*m*, *w*, *l*) *P *(*d*)

where Pr(gap ≤ *g*|*m*, *w*, *l*) is given by Equation 3 and,

P(d)=∏i=1md12,     (6)
 MathType@MTEF@5@5@+=feaafiart1ev1aaatCvAUfKttLearuWrP9MDH5MBPbIqV92AaeXatLxBI9gBaebbnrfifHhDYfgasaacH8akY=wiFfYdH8Gipec8Eeeu0xXdbba9frFj0=OqFfea0dXdd9vqai=hGuQ8kuc9pgc9s8qqaq=dirpe0xb9q8qiLsFr0=vr0=vr0dc8meaabaqaciaacaGaaeqabaqabeGadaaakeaacqWGqbaucqGGOaakcqWGKbazcqGGPaqkcqGH9aqpdaqeWbqaamaalaaabaGaeGymaedabaGaeGOmaidaaaWcbaGaemyAaKMaeyypa0JaeGymaedabaGaemyBa02aaSbaaWqaaiabdsgaKbqabaaaniabg+GivdGccqGGSaalcaWLjaGaaCzcamaabmaabaGaeGOnaydacaGLOaGaayzkaaaaaa@40E4@

where *m*_*d *_is the number of motifs for which strand information is known.

#### Combining significance values from individual sites and module structure

With *p*-values calculated for individual sites as well as for the clustering property of the module, we can combine these significance values to get an overall module significance. There are a number of ways that this can be done depending on the situation. If it is desirable to have the individual sites contribute more to the overall module score, the *p*-values for the individual sites can be weighted accordingly. Conversely, if the clustering property is more important, the probability of clustering can be weighted more. The most straightforward way to combine the *p*-values is just to take the product of the individual *p*-values as the overall probability value and apply a Bonferroni correction. The technique we choose to combine the *p*-values in MODSTORM is the technique used by Bailey and Gribskov [48].

#### Scanning for the IFN-*β *enhancer

**Figure 4:**
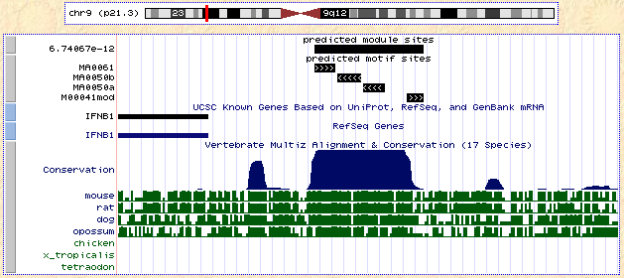
**IFN-*β *enhancer**. A screen shot of the IFN-*β *enhancer found with MODSTORM as a track on the UCSC genome browser (Human Mar. 2006 (hg18) assembly [55]). The top track spans the entire length of the module and is labeled with the module significance. The individual motifs occurrences are shown below. The predicted location of this site is consistent with the experimentally verified site.

The IFN-*β *enhancer is a classic example of an module with organizational constraints, occurring in the promoter of the IFN-*β *gene [49,50]. To examine the ability of MODSTORM to find modules with organization in the human genome, we scanned all intergenic regions in the human genome with a module representing the IFN-*β *enhancer (for details see Additional file 2). Scanning for this module using MODSTORM and a module without any constrained organizational parameters finds nine occurrences of this module genome-wide. By enforcing the organization, only one occurrence is found and it is immediately upstream of the IFN-*β *gene. The location of this predicted module is shown in Figure 4. The *p*-value for this module site is 6.74e-12. This module site is also found when searching for the module without defined organizational parameters. However, the module *p*-value in this case is only 2.13e-9.

To compare MODSTORM with other existing module scanning tools, we scanned for the IFN-*β *module in a sequence set consisting of all large promoters (-5000 to +500 w.r.t. TSS). The MODSTORM software, with or without organization, scans through all of these sequences and finds the one true occurrence of this module in roughly two minutes and 30 seconds on a modern LINUX workstation. CisModScan takes 30 minutes on the same machine to repeat the same task and is not able to find the true module site. Sosinsky *et al*. tested Target Explorer on the task of finding the IFN-*β *module. Target Explorer was used to scan for windows of 50 bp within which at least one binding site for c-Jun, ATF-2, IRF, and NF-*κ*B occurred and it was able to find a window where all occurred in the human interferon-beta promoter [46]. Target Explorer does not attempt to assign any statistical significance to module occurrences. Aerts *et al*. also applied the ModuleScanner algorithm to the problem of finding the IFN-*β *enhancer. They constructed a module consisting of motifs for NF-*κ*B, IRF-1 and HMGIY. ModuleScanner found many occurrences of this module, with the fourth most significant hit being upstream of the human interferon-beta gene. In general, algorithms that just scan for module occurrences will be much faster than any of the algorithms that attempt to cluster together single sites from a library of known matrices. To illustrate this, we tested the COMET software for its ability to find the IFN-*β *enhancer. COMET, given the four PFMs that make up the IFN-*β *module as a library, scans for two hours, again on the same machine. While it does find some clusters containing three or four matrices, COMET does not find the true site.

#### Implementation of STORM and MODSTORM

The method of using a sequence database to convert between score cutoffs and *p*-values was implemented in STORM, the Search Tool for Occurrences of Regulatory Motifs. STORM searches sequences for sites that match a scoring matrix, and is ideally suited for searching for multiple motifs simultaneously because the sequences are preprocessed as suffix trees [51]. Once the suffix trees are constructed, scoring matrices are matched along the suffix trees using a branch and bound strategy, making the time to search with each matrix proportional to the width of the matrix, instead of the length of the sequence.

The MODSTORM software preprocesses sequences into suffix trees in the same manner as STORM. When searching for modules without organizational constraints, the motifs making up the module are ranked by information content. The highest information content motif is scanned for first. If occurrences of this motif are found, the remaining motifs are searched for in a window surrounding the first located motif. The motifs are searched for in order of information content. If at any step, the motif being searched for is not found in the window, the search for the remaining factors is abandoned and the scanning for the highest information content motif is resumed.

When searching for modules with organizational constraints, the 5' most motif is scanned for first. If an occurrence of this is detected, a window size defined by the organization of the module is scanned for the next motif in the module and so on. If any one of the motifs is not discovered, the scanning for the 5' most motif is resumed.

## Discussion

### Importance of choosing the correct database to build (*g*, *k*)-tables

For *p*-values to be informative they must be calculated relative to the appropriate sequence database. When searching for sites in 1 kb proximal promoters from the rat genome, the most appropriate database to use is the complete set of 1 kb rat promoters. When scanning through human intergenic regions that have been masked using RepeatMasker [52], the most appropriate background sequence set to use in constructing a (g, k)-table is the complete set of masked human intergenic regions. One common problem in transcription factor binding site detection is the presence of CpG islands. To address this issue, tables can be constructed for CpG and non-CpG sequence and the appropriate table can be used when scanning in regions where the CpG status is known. In addition to CpG islands, there are numerous features of genomic sequence (such as repetitive regions) that could affect the statistics calculated using (g, k)-tables. The selection of a proper background set to create (*g*, *k*)-tables when analyzing such regions is imperative to the interpretation of the results. In addition to the software which creates (*g*, *k*)-tables, we provide tables calculated for human, mouse and rat promoters from the CSHL mammalian promoter database for three sizes: small (-500 to +100 w.r.t TSS), medium (-1000 to +300 w.r.t. TSS) and large (-5000 to +500 w.r.t. TSS) as well as tables constructed from all intergenic regions in human, mouse and rat. It is important to stress the difference between statistical and biological significance of k-mers in genomic sequence. While this method will find k-mers that are statistically significant, wet lab experiments must be performed to determine biological significance.

### Finding optimal modules with organizational parameters

When scanning for modules with organizational parameters, the current method will not necessarily find the optimal scoring module. A scenario exists where weaker motifs existing in advantageous organization would score higher than stronger motifs in less advantageous organization in the same genomic window. Our algorithm is based on finding motifs first and then assessing organization and would thus return the stronger motifs with the weaker organization as the module hit from this region.

Modifications of our current algorithm which performs the module search in a more iterative fashion would be better able to deal with the situation of weaker motifs having strong organization. For example, after finding module sites, the threshold scores of the motifs comprising the module could be lowered and the module region could be searched for each motif again. If lower scoring motifs are found, the module could be optimized by its overall significance by considering motif scores and organization.

### Extension of MODSTORM to module discovery

The MODSTORM software could easily be extended to library based module discovery algorithm. In this case, a library of known motifs would be scanned across every window and motif combinations that lead to significant modules would be retained. This is an interesting problem and there are multiple existing software tools that attempt to solve this problem, several of which have been mentioned above. We choose to focus here on the efficient scanning for defined modules as this is an area of research that is lacking.

## Conclusion

We have introduced novel methods to aid in the detection and estimation of statistical significance for both single sites and modules of transcription factor binding sites. Our method of using real promoters to estimate statistical parameters provides an intuitive way to interpret the threshold for binding site matches. The techniques introduced to calculate the statistical significance of clusters of transcription factor binding sites allow for the statistical evaluation of putative *cis*-regulatory modules. The search algorithms and statistical significance calculations implemented in the software tools we provide allow for the scanning of large intergenic sequences for single transcription factor binding sites as well as *cis*-regulatory modules. These software tools have the potential to greatly aid in the *in silico *study of transcriptional regulation.

## Methods

### Other scoring methods allowed in STORM and MODSTORM

In addition to scoring putative motif site matches with *p*-values as described above, we have also incorporated a number of other scoring schemes into the STORM and MODSTORM software. The additional scoring methods include standard PWM threshold scoring, the percentage of maximum score, and the functional depth.

A common and intuitive approach for cutoff selection takes the maximum possible score derived from a given matrix, and sets the threshold for occurrence at some percentage of the maximum score. This idea was shown to have merit by Tronche *et al*., [53] who showed that HNF1 sites with a score greater than 83% of the maximum score of the scoring matrix showed experimental evidence of binding. Although this score of 83% of the maximum score works well for HNF1, it is expected that different factors will have different percentages of maximum scores allowed for binding.

The term "functional depth" was first introduced by Beer and Tavazoie [54] as a term to represent the threshold above which functional binding will occur for a particular factor represented by a PWM. We define the functional depth for a PWM score S as,

functional depth=S−SminSmax−Smin
 MathType@MTEF@5@5@+=feaafiart1ev1aaatCvAUfKttLearuWrP9MDH5MBPbIqV92AaeXatLxBI9gBaebbnrfifHhDYfgasaacH8akY=wiFfYdH8Gipec8Eeeu0xXdbba9frFj0=OqFfea0dXdd9vqai=hGuQ8kuc9pgc9s8qqaq=dirpe0xb9q8qiLsFr0=vr0=vr0dc8meaabaqaciaacaGaaeqabaqabeGadaaakeaacqqGMbGzcqqG1bqDcqqGUbGBcqqGJbWycqqG0baDcqqGPbqAcqqGVbWBcqqGUbGBcqqGHbqycqqGSbaBcqqGGaaicqqGKbazcqqGLbqzcqqGWbaCcqqG0baDcqqGObaAcqGH9aqpdaWcaaqaaiabdofatjabgkHiTiabdofatnaaBaaaleaaieGacqWFTbqBcqWFPbqAcqWFUbGBaeqaaaGcbaGaem4uam1aaSbaaSqaaiab=1gaTjab=fgaHjab=Hha4bqabaGccqGHsislcqWGtbWudaWgaaWcbaGae8xBa0Mae8xAaKMae8NBa4gabeaaaaaaaa@5684@

where *S*_*min *_and *S*_*max *_are the minimum and maximum scores possible for the PWM.

## Availability and requirements

Project name: STORM

Project home page: 

Operating system(s): Linux/Unix

Programming language: C++

Other requirements: The STORM and MODSTORM programs are available as part of the Comprehensive Regulatory Element Analysis and Discovery (CREAD) package, see  for more information.

License: GNU GPL

Any restrictions to use by non-academics: None

## Authors' contributions

DS conducted the experiments; DS and AS contributed to writing of software; all authors contributed to design of the methodology and writing of the manuscript.

## Supplementary Material

Additional file 1 Appendix. The number of ways to place *m *motifs with widths in a sequence of length *l*. Click here for file

Additional file 2 IFN-*β *Enhancer. Details on the module used to scan for the IFN-*β *enhancer. Click here for file
