# Supplementary material for: Statistical significance of cis-regulatory modules
Source: BMC Bioinformatics. 2007 Jan 22;8:19. doi: 10.1186/1471-2105-8-19 (PMC1796902; doi:10.1186/1471-2105-8-19)
Supplement: Additional file 1 — Appendix. The number of ways to place m motifs with widths in a sequence of length l. [file 1471-2105-8-19-S1.pdf]

APPENDIX

A The number of ways to place m motifs with widths in a sequence of length l without overlaps

We show here the number of ways to place $m = 2$ motifs with widths in a sequence of length l with no overlaps. The general case can be proved by induction on the number of motifs.

Proof The number of ways to place two motifs, M_A of width w_A and M_B of width w_B in a sequence of length l is

$$\psi(l, w_A, w_B, 2) = (\text{number of ways to place } M_A) \times (\text{number of ways to place } M_B \text{ given } M_A \text{ placement}). \quad (1)$$

Most placements of M_A will prevent the placement of M_B at w_A positions, the positions occupied by M_A. The exceptions to this situation occur when M_A is within $w_B - 1$ of either the beginning or end of the sequence. When M_A is placed at position i where $0 \le i \le w_B - 1$ or $l - (w_A - 1) - w_B + 1 \le i \le l - (w_A - 1)$, the placement of M_B is constrained. In addition to not being able to be placed on the w_A positions M_A is occupying, it further is not able to be placed between M_A and the closest end of the sequence. We refer to the cases of M_A starting at $\{0, \dots, w_B - 1\}$ and $\{l - (w_A - 1) - w_B + 1, \dots, l - (w_A - 1)\}$ as *exceptional* cases, with the other cases refereed to as *regular* cases.

With this division, we can now write

$$\psi(l, w, 2) = (\text{number of regular placements of motif } M_A) \times (\text{placements of } M_B \text{ given that } M_A \text{ has a regular placement}) + \sum_{i=1}^{w-1} (\text{placements of } M_B \text{ given } M_A \text{ has exceptional placement } i). \quad (2)$$

The first term in Equation 2 can be written as

$$\Big(l - w_B - w_B - (w_A - 1)\Big)\Big(l - w_A - (w_B - 1) - (w_B - 1)\Big) \quad (3)$$

The second term, summing over the *exceptional* cases can be written as

$$2 \times \sum_{i=0}^{w_B - 1} \Big[l - (w_B - 1) - w_A - i\Big], \quad (4)$$

where factor of 2 is to deal with the *exceptional* cases both in the beginning and the end of the sequence. Substituting Equations 3 and 4 into Equation 2, we have

$$\psi(l, w_A, w_B, 2) = \Big[l - (w_A - 1) - 2w_B\Big]\Big[l - w_A - 2(w_B - 1)\Big] + 2 \times \sum_{i=0}^{w-1} \Big[l - (w_B - 1) - w_A - i\Big].$$

It is straightforward to show that this is equivalent to

$$2 \times \binom{L - \sum_i (w-1)}{2}.$$

This completes the proof for the $m = 2$ case. ∎
