# Supplementary material for: Statistical significance of cis-regulatory modules
Source: BMC Bioinformatics. 2007 Jan 22;8:19. doi: 10.1186/1471-2105-8-19 (PMC1796902; doi:10.1186/1471-2105-8-19)
Supplement: Additional file 2 — IFN-β Enhancer. Details on the module used to scan for the IFN-β enhancer. [file 1471-2105-8-19-S2.pdf]

IFN-β Enhancer

The IFN-β enhancer consists of binding sites for HMGI, ATF-2/c-Jun, IRF-1 and NF-κB proteins [1, 2, 3]. The binding sites for many of these factors are overlapping in the module, however, so we chose to construct a module out of four motif profiles: a modified ATF-2/c-Jun profile, two IRF-1 profiles and an NF-κB profile. The modified ATF-2/c-Jun profile was created by combining a profile for c-Jun in TRANSFAC [4] (M00041) with a profile for ATF-2 in TRANSFAC (M00040). The two IRF-1 profiles were constructed by modifying a profile for IRF in JASPAR [5] (MA0050). The profile used for NF-κB was from JASPAR (MA0061). The order of the motifs was selected as the order reported by Thanos [1]. The spacings between the individual factors as well as the individual motif p-values were learned on the human IFN-B promoter. A cartoon of this module is shown in Figure 1. The module file that was used is also given at http://rulai.cshl.edu/storm/SupplementaryMaterial.

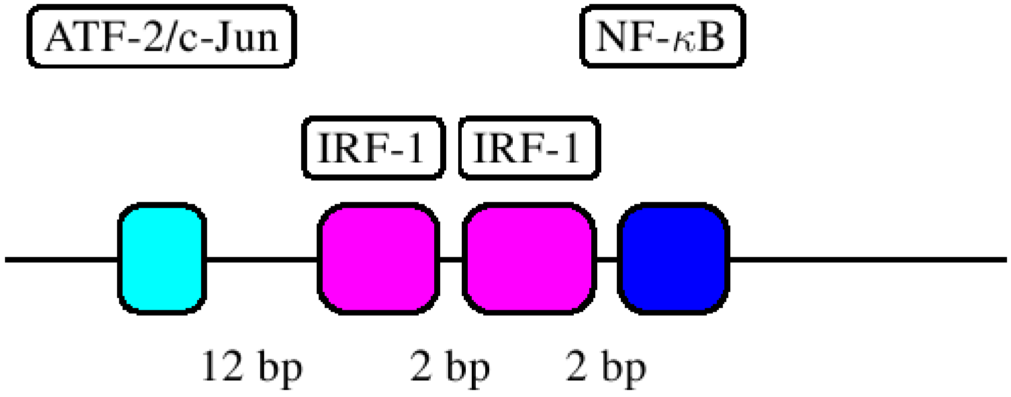

Figure 1: The module used to scan for IFN-β occurrences. Consists of a modified ATF-2/c-Jun profile, two IRF-1 profiles and an NF-κB profile. The distance allowed between any two sites are listed below the spacings.

The (g, k)-table used was built from all human promoters from the CSHL mammalian promoter database with sequence spanning -500 to +100 w.r.t TSS, with $k = 6$ and $g = 5$.

References

[1] Thanos D: **Mechanisms of Transcriptional Synergism of Eukaryotic Genes, The Interferon- Paradigm**. *Hypertension* 1996, **27**:1025–1029.

[2] Struhl K: **A Paradigm for Precision**. *Science* 2001, **293**:1054–1055.

[3] Munshi N, Yie J, Senger K, Lomvardas S, Agalioti T, Thanos D: **The IFN-β Enhancer: A Paradigm for Understanding Activation and Repression of Inducible Gene Expression**. *Cold Spring Harbor Symposia on Quantitative Biology* 1999, **LXIV**:149–159.

[4] Matys V, Kel-Margoulis OV, Fricke E, Liebich I, Land S, Barre-Dirrie A, Reuter I, Chekmenev D, Krull M, Hornischer K, Voss N, Stegmaier P, Lewicki-Potapov B, Saxel H, Kel AE, E W: **TRANSFAC and its module TRANSComple: transcriptional gene regulation in eukaryotes**. *Nucleic Acids Research* 2006, **34**:D108–110.

[5] Sandelin A, Alkema W, Engström P, Wasserman WW, Lenhard B: **JASPAR: an open access database for eukaryotic transcription factor binding profiles**. *Nucleic Acids Research* 2004, **32**:D91–D94.
